# A comparative study of asleep and awake deep brain stimulation robot-assisted surgery for Parkinson’s disease

**DOI:** 10.1038/s41531-020-00130-1

**Published:** 2020-10-05

**Authors:** Hai Jin, Shun Gong, Yingqun Tao, Hua Huo, Xiao Sun, Dandan Song, Ming Xu, Zhaozhu Xu, Yang Liu, Shimiao Wang, Lijia Yuan, Tingting Wang, Weilong Song, He Pan

**Affiliations:** 1 Department of Neurosurgery, The General Hospital of Northern Theater Command, 83 Wenhua Road, 110016 Shenyang, China; 2 Department of Clinical Trail Management Agency, The General Hospital of Northern Theater Command, 83 Wenhua Road, 110016 Shenyang, China; 3 Department of Anesthesia, The General Hospital of Northern Theater Command, 83 Wenhua Road, 110016 Shenyang, China

**Keywords:** Parkinson's disease, Parkinson's disease

## Abstract

To compare the differences between asleep and awake robot-assisted deep brain stimulation (DBS) surgery for Parkinson’s Disease (PD), we conducted this retrospective cohort study included 153 PD patients undergoing bilateral robot-assisted DBS from June 2017 to August 2019, of which 58 cases were performed under general anesthesia (GA) and 95 cases under local anesthesia (LA). Procedure duration, stimulation parameters, electrode implantation accuracy, intracranial air, intraoperative electrophysiological signal length, complications, and Unified PD Rating Scale (UPDRS) measurements were recorded and compared. The clinical evaluation was conducted by two raters who were blinded to the choice of anesthesia. Procedure duration was significantly shorter in the GA group, while on stimulation off medication motor scores (UPDRS-III) were significantly improved in both the GA and LA group. ANCOVA covariated for the baseline UPDRS-III and levodopa challenge exhibited no significant differences. In terms of amplitude, frequency, and pulse width, the stimulation parameters used for DBS power-on were similar. There were no significant differences in electrode implantation accuracy, intraoperative electrophysiological signal length, or intracerebral hemorrhage (no occurrences in either group). The pneumocephalus volume was significantly smaller in the GA group. Six patients exhibited transient throat discomfort associated with tracheal intubation in the GA group. The occurrence of surgical incision infection was similar in both groups. Compared with the awake group, the asleep group exhibited a shorter procedure duration with a similar electrode implantation accuracy and short-term motor improvement. Robot-assisted asleep DBS surgery is a promising surgical method for PD.

## Introduction

Deep brain stimulation (DBS) surgery is an effective treatment for Parkinson’s disease (PD)^1,2^. To date, awake surgery has been typically performed with intraoperative test stimulations^3^. Recently, asleep surgery has been performed under general anesthesia (GA) with intraoperative magnetic resonance imaging (iMRI)^4^ or computed tomography (iCT)^5^ to confirm the position of the electrode tip. However, limited research and clinical experience has been reported regarding robot-assisted asleep surgery. Herein, this study reports robot-assisted DBS surgical procedures and clinical results in detail.

## Results

### General data

In total, 153 patients were retrospectively included in this study, with 58 cases in the GA group and 95 cases in the LA group. The mean ages were 63.5 ± 6.6 years (GA group) and 63.2 ± 9.7 years (LA group). Males represented 37.9% (22/58) of the GA group and 58.9% (56/95) of the LA group. The disease durations were 10.5 ± 5.0 years (GA group) and 8.7 ± 4.6 years (LA group). The Hoehn & Yahr (H & Y) stages were 3.01 ± 0.27 (GA group) and 2.99 ± 0.23 (LA group). The preoperative UPDRS scores (OFF) were 63.8 ± 13.6 (GA group) and 62.2 ± 16.3 (LA group). The preoperative UPDRS-III scores (OFF) were 28.9 ± 6.9 (GA group) and 28.6 ± 9.0 (LA group), and the preoperative UPDRS-III scores (ON) were 14.3 ± 6.6 (GA group) and 14.8 ± 7.3 (LA group). The preoperative levodopa daily doses were 652.4 ± 400.3 mg/d (GA group) and 577.6 ± 340.8 mg/d (LA group). The preoperative levodopa responses (UPDRS-III improvement) were 50.0 ± 17.8 % (GA group) and 48.8 ± 16.2% (LA group). There were no significant differences in the baseline values between the two groups apart from sex and disease duration (Table 1).

**Table 1:** Demographic and clinical characteristics of the PD patients.

Characteristic	GA group (*n* = 58)	LA group (*n* = 95)	*P* value
Age-year	63.5 ± 6.6	63.2 ± 9.7	0.8710
Male sex-no. (%)	22 (37.9)	56 (58.9)	0.0116
Disease duration - Y	10.5 ± 5.0	8.7 ± 4.6	0.0304
H & Y stage	3.01 ± 0.27	2.99 ± 0.23	0.6429
UPDRS scores (Med OFF)	63.8 ± 13.6	62.2 ± 16.3	0.5352
UPDRS-III scores (Med OFF)	28.9 ± 6.9	28.6 ± 9.0‘	0.8383
UPDRS-III scores (Med ON)	14.3 ± 6.6	14.8 ± 7.3	0.6795
Levodopa daily dose (mg/d)	652.4 ± 400.3	577.6 ± 340.8	0.2197
Levodopa response (UPDRS-III improvement) (%)	50.0 ± 17.8	48.8 ± 16.2	0.6649
MoCA scores	26.7 ± 1.6	27.2 ± 1.3	0.0501
PDQL-39 scores	86.6 ± 10.4	90.2 ± 11.6	0.0547
DBS target-STN vs Gpi	38 vs. 20	61 vs. 34	0.8697

*PD* Parkinson’s disease, *DBS* deep brain stimulation, *STN* subthalamic nucleus, *Gpi* globus pallidus internus, *Y* year, *UPDRS* Unified Parkinson’s disease Rating Scales, *MoCA* Montreal Cognitive Assessment Scale, *PDQL-39* PD quality of life, *H & Y stage* Hoehn & Yahr stage.

### Procedure duration, pneumocephalus volume, levodopa equivalent of daily dose reduction, and Tao’s DBS surgery scale

**Fig. 1:**
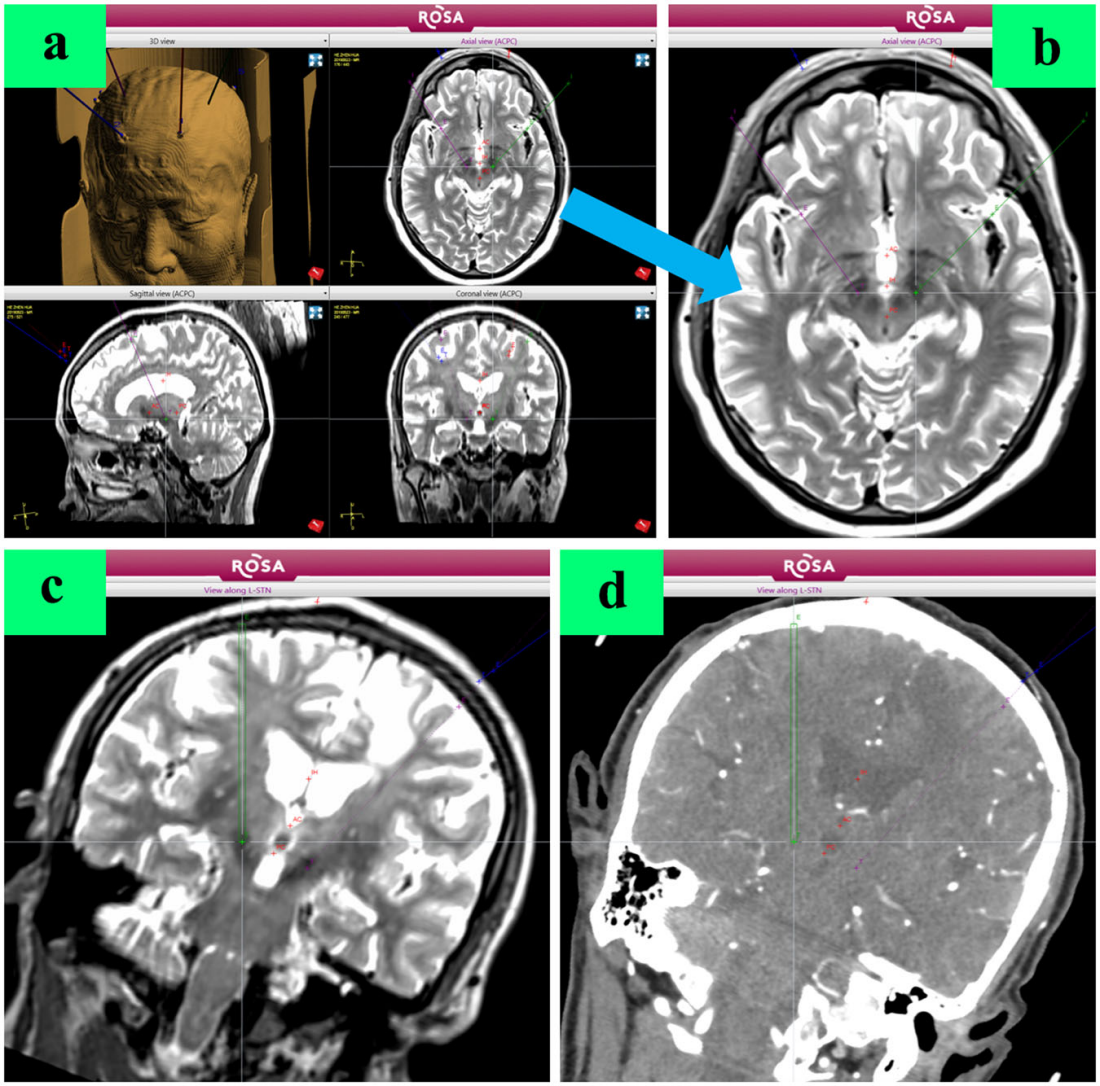
Preoperative planning. **a** DBS surgical planning in ROSA Robot system. **b** target shown in MRI T2 image. **c** Trajectory planning relying on MRI image. **d** Trajectory planning relying on contrast-enhanced CT image to avoid damage to cerebral vessels.

All 306 electrodes of the 153 cases were implanted successfully without adjustment of the electrode position during the ROSA robot-assisted surgery. The duration of the procedure was significantly shorter in the GA group (1.09 ± 0.46 h) than in the LA group (1.54 ± 0.57 h, *p* < 0.0001). The total MER time was significantly shorter in the GA group (12.03 ± 1.77 min) than in the LA group (12.89 ± 2.73 min, *p* = 0.0338). There was no significant difference in the levodopa equivalent of daily dose reduction between the two groups (162.8 ± 431.6 mg (GA) vs. 208.0 ± 403.5 mg (LA), *p* = 0.5141). The pneumocephalus volume was significantly smaller in the GA group (4.35 ± 5.61 cm^3^) than in the LA group (12.25 ± 13.76 cm^3^, *p* < 0.0001). The Tao’s DBS surgery scale^6^ was significantly higher in the GA group (85.2 ± 9.3) than in the LA group (76.5 ± 8.0, *p* < 0.0001, Fig. 1).

### Electrode implantation accuracy

The electrode implantation accuracy did not differ between the GA group (0.71 ± 0.25 mm) and LA group (0.76 ± 0.23 mm, *p* = 0.3031). The intraoperative electrophysiological signal length also did not differ between the groups (for STN, 5.90 ± 1.45 mm (GA) vs. 5.47 ± 0.82 mm (LA), *p* = 0.0630; for Gpi, 8.68 ± 2.52 mm (GA) vs. 9.48 ± 2.39 mm (LA), *p* = 0.2525).

### UPDRS and UPDRS-III

The on stimulation off medication UPDRS scores significantly improved in both groups (GA: 26.84 ± 12.23, *p* < 0.0001; LA: 25.87 ± 12.59, *p* < 0.0001). ANCOVA covariated for the baseline UPDRS and levodopa challenge revealed no significant differences (ANCOVA *F* = 0.009, Bonferroni *p* = 0.923). The percentage improvement was similar in both groups (42.8 ± 19.0% (GA) and 41.6 ± 15.9% (LA), ANCOVA F = 0.163, Bonferroni *p* = 0.687). The on stimulation off medication motor scores (UPDRS-III) significantly improved in both group GA (18.31 ± 6.83, *p* < 0.0001) and LA (16.24 ± 7.14, *p* < 0.0001). ANCOVA covariated for the baseline UPDRS-III and levodopa challenge revealed no significant differences (ANCOVA *F* = 1.233, Bonferroni *p* = 0.269). The percentage improvement was similar in both groups (GA: 63.4 ± 19.2%; LA: 57.0 ± 17.3%; ANCOVA *F* = 0.718, Bonferroni *p* = 0.398).

### Stimulation parameters

In terms of amplitude, frequency, and pulse width, the stimulation parameters used for DBS power-on were similar (*p* > 0.05). The amplitude stimulation parameter used for neuromodulation was significantly lower in the GA-STN group (2.33 ± 0.62 V) than in the LA-STN group (2.77 ± 0.70 V, *p* = 0.0018), yet not significantly different between the GA-GPi (3.94 ± 0.45 V) and LA-GPi groups (3.73 ± 0.70 V, *p* = 0.2504, Table 2).

**Table 2:** Comparison of the stimulation parameters between two groups.

DBS targets	Characteristic	GA	LA	*P* value
STN (GA = 38, LA = 61)	Mean amplitude (V)	1.47 ± 0.42	1.47 ± 0.51	0.9832
	Mean frequency (Hz)	145.0 ± 21.5	136.5 ± 22.8	0.0698
	Mean pulse width (μs)	62.1 ± 5.2	61.3 ± 8.5	0.6054
	Amplitude stimulation for neuromodulation (V)	2.33 ± 0.62	2.77 ± 0.70	0.0018
Gpi (GA = 20, LA = 34)	Mean amplitude (V)	1.96 ± 0.46	1.72 ± 0.43	0.0574
	Mean frequency (Hz)	134.0 ± 24.2	137.1 ± 24.8	0.6602
	Mean pulse width (μs)	65.5 ± 9.6	68.5 ± 9.2	0.2542
	Amplitude stimulation for neuromodulation (V)	3.94 ± 0.45	3.73 ± 0.70	0.2504

*DBS* deep brain stimulation, *STN* subthalamic nucleus, *Gpi* globus pallidus internus, *GA* general anesthesia, *LA* local anesthesia.

### Postoperative side effects and complication rates

The postoperative side effects during neuromodulation included muscle contraction (*n* = 7), dysarthria (*n* = 1), oculomotor dysfunction (*n* = 12), dysesthesia (*n* = 1), dizziness (*n* = 23), palpitation (*n* = 10), and dyskinesia (*n* = 27) in the GA group and muscle contraction (*n* = 11), dysarthria (*n* = 2), oculomotor dysfunction (*n* = 27), dysesthesia (*n* = 11), dizziness (*n* = 48), palpitation (*n* = 3), and dyskinesia (*n* = 34) in the LA group.

In the LA group, one participant (a 65-year-old male) was allergic to lidocaine. After local administration of lidocaine, the patient immediately exhibited dyspnea, loss of consciousness, and decreased blood pressure. The subsequent rescue was successful. Because of the precise positioning of the robot and fine surgical operations, no intracranial hematomas occurred in any of the cases. Incision infection was similar in both groups (two cases in the GA and two cases in the LA group). In the GA group, one case (a 71-year-old female) exhibited rejection such as swelling and discomfort at the site of skin incision and six patients exhibited transient throat discomfort associated with tracheal intubation.

## Discussion

In the last 30 years, awake surgery has been widely performed^7^, most often under LA with intraoperative test stimulation^3^. During awake surgery and without intraoperative imaging, neurosurgeons determine electrode placement by relying on electrophysiological typical signals and the patients’ symptom relief without any side effects. In recent years, there have been some reports on the use of intraoperative imaging of DBS under GA. In our current study, we report the application of a robot for DBS of PD patients under GA, with which we achieved good results.

The main difference between asleep and awake DBS surgery is the method of intraoperatively verifying the position of electrode implantation. Some neurosurgical centers have performed asleep DBS surgery under GA with iMRI^4,8,9^ or iCT merged together with preoperative MRI to verify the accuracy of electrode implantation^10,11^. It has been demonstrated that the clinical outcomes and complication rates of asleep surgery are comparable to those in historical studies using MER to guide or confirm lead placement under LA^12–15^. An advantage of iCT or iMRI guidance is the ability to account for brain shift following dural opening and cerebrospinal fluid loss^16^. The ideal intraoperative imaging modality for use during asleep DBS surgery remains to be confirmed, and further data are needed to provide accurate comparisons between the outcomes of iCT and iMRI. The disadvantages of iCT and iMRI include longer surgeries and longer times under anesthesia as well as higher risks related to imaging procedures and inevitable errors. The most important disadvantage of iCT and iMRI is that intraoperative images under pneumocephalus or loss of cerebrospinal fluid can merely reflect the real electrode position. The accuracy obtained by merged iMRI or iCT data cannot reflect the real accuracy, which can only be confirmed in postoperative images (after 1–2 weeks) after the brain shift resolves.

Electrode implantation accuracy is a key point related to the prognosis of DBS surgery. The procedures of awake and asleep DBS surgeries rely upon different methods for the verification of the intended target acquisition. There are many factors that affect accuracy, such as head position^17^, surgical procedures, pneumocephalus, and brain shift. In our study, the electrode implantation accuracy was 0.71 ± 0.25 mm in the GA group and 0.76 ± 0.23 mm in the LA group. Our results indicate that asleep DBS robot-assisted surgery can provide adequate accuracy. In our experience, the specific surgical procedures ensured accuracy by using a modified registration, intraoperative registration, and simulated target verification^6,18^. Furthermore, intraoperative MER was used to confirm the final position of the electrode.

Despite the support of the above surgical techniques, we used a single-channel microelectrode to record the electrophysiological signals and thus to confirm the target location. Under GA monitored by BIS, many factors can interfere with the MER signal. Sometimes, there was even no MER signal at all. Previously, we reduced the depth of GA. When the BIS value score is >70, BIS monitoring is turned off to avoid interference and to thus obtain a typical MER signal. Once no signal can be recorded, the accuracy of electrode implantation is verified by the merged result of 2 h of postoperative CT and preoperative planning images. Of note, in our 153 cases no adjustment for electrode position was required during surgery.

A meta-analysis found that there were no significant differences between the clinical outcomes of the MER and non-MER groups^17^. In a retrospective study, the mean (±SD) duration of the procedure for all 323 cases under iCT was 2.51 ± 1.04 h^19^. Mirzadeh et al.^19^ found that the MER was an independent contributor to increased procedure durations (+44 min) and that improved accuracy was associated with shorter procedure durations. In our study, the procedure duration was significantly shorter in the GA group. On the basis of the above results, a possible ideal asleep DBS surgery may be performed without MER and intraoperative imaging to reduce excessive surgical procedures. Such an ideal asleep DBS surgery is most likely to be achieved under the following conditions: correction registration, intraoperative registration, and simulated target verification of the robot. Such surgery should be performed by skilled neurosurgeons and their teams. Nevertheless, for this type of surgery to be approved, more prospective clinical studies are necessary.

Most studies that investigated outcomes after DBS asleep surgery and that found comparable results to awake surgery had no control group but rather compared their outcomes with previous studies^8,13,20,21^. In a retrospective study, Tsai et al.^22^ found that, in terms of UPDRS score improvement, levodopa equivalent of daily dose reduction and stimulation parameters did not show significant differences between groups after 5 years. Blasberg et al.^13^ found a significant difference in the percentage reduction of UPDRS-III motor scores due to stimulation after 3 months but not after 1 year. Using the baseline values of UPDRS and levodopa challenge as covariates, we found that the short-term UPDRS-III improvement rate in the GA group was similar to that of the LA group.

The short-term clinical outcomes in this study were consistent with our previous findings regarding the DBS surgical scoring method (Tao’s DBS surgery scale)^6^. The scale of asleep DBS surgery was higher than that of awake DBS surgery in this study. Although patients undergo extensive pre- and postoperative evaluation, the field lacks a robust scoring system for quantifying DBS surgery. To determine whether a practical scale could assess DBS surgery and predict its clinical significance, we designed the Tao’s DBS surgery scale. The scale draws upon multi-factor statistical analysis of factors that affect the efficacy of DBS surgery in patients with PD and was designed to evaluate the quality of DBS surgery, as well as to help improve its efficacy. It consists of the following parts: electrode implantation duration, postoperative pneumocephalus volume, and electrode fusion error. At present, it is derived from single-center data and thus requires further research and verification.

The current study demonstrated no significant differences in postoperative “freezing” and “speech” between groups. The compared clinical outcome statements still require further observation and long-term follow-up of motor/non-motor and neuropsychological symptoms and side effects (e.g., cognitive, mood, and behavioral effects). Taken together, although most of these studies showed comparable results for both procedures, they were limited because of the absence of control groups or at best unmatched groups with different baseline characteristics, small sample sizes for direct and matched comparisons, or short postoperative observation periods ^13^.

The literature on the impact of complications associated with the use of GA during asleep DBS surgery is limited. Recent studies^15,17,23,24^ have demonstrated that the incidence of intracerebral hemorrhages, infections, and epilepsy were similar between asleep and awake DBS surgery, which is generally consistent with our results. In this study, the volume of intracranial air was significantly lower in the GA group than that in the LA group, which is consistent with previous reports^17,25^. Awake DBS resulted in significantly larger cortical brain shifts^25^. Additionally, awake DBS surgery has the disadvantage of potential local anesthetic drug allergies^26^.

A literature review^20^ published recently revealed that there are no significant differences in cost between awake and asleep DBS surgery. In contrast, in a single academic medical center cost analysis, asleep DBS surgery was associated with lower costs in comparison with the awake procedures^27^. The cost was influenced by the use of iCT, iMRI, or a robot, as well as anesthesia-related expenses and postoperative incidents or complications.

Certain limitations were present in our study design. First, this study was a retrospective cohort study, which limits its external validity. Nevertheless, the patients in the two groups were consecutively recruited and returned to all postoperative follow-ups. There was no randomization for the group assignment (asleep vs. awake), which implies a selection bias. Second, we investigated robot-assisted asleep DBS surgery for PD in a single center, even though this technique is still not widely used. We insist that this is a DBS surgical method with future prospects. Third, the follow-up time for UPDRS score evaluation (including subscale scores) was 6 months on average. Long-term follow-ups are still needed for both groups. The lack of good-quality randomized clinical trials warrants further research in this field.

Compared with the awake group, the asleep group exhibited a shorter procedure duration and a similar electrode implantation accuracy and short-term motor improvement. In general, DBS surgery should still be performed with the technique that the neurosurgeon and team members are most familiar with, because this provides the patients with the best possible outcome. Robot-assisted asleep DBS surgery is a promising surgical method for PD in the future. However, high-quality epidemiological data are lacking. Thus, a prospective randomized controlled trial with a larger patient population and longer follow-up is needed to confirm the findings and conclusions of this study.

## Methods

### Patient selection

All PD patients who underwent bilateral subthalamic nucleus (STN) or globus pallidus internus (Gpi) DBS surgery from June 2017 to August 2019 at the General Hospital of Northern Theater Command were included. These patients met the diagnostic criteria of the United Kingdom PD Brain Bank, in which at least two of the cardinal symptoms were present. Before surgery, each patient underwent a levodopa test to ensure a positive levodopa response (Unified PD Rating Scale (UPDRS) part III >30% improvement in scores). The same DBS team, with one senior neurosurgeon (Professor YQ Tao) and one senior anesthesiologist (Doctor DD Song), performed all DBS procedures for these patients at our hospital. The surgical procedures have been described in our previous reports^6,18^. Because of the lack of DBS surgery guidelines regarding GA or local anesthesia (LA), this choice was made by the patient after we informed the patients and their families about the potential benefits and risks of both GA and LA. The clinical evaluation was conducted by two raters (Doctors Yang Liu and Shimiao Wang) who were blinded to the choice of anesthesia.

### Preoperative planning

**Fig. 2:**
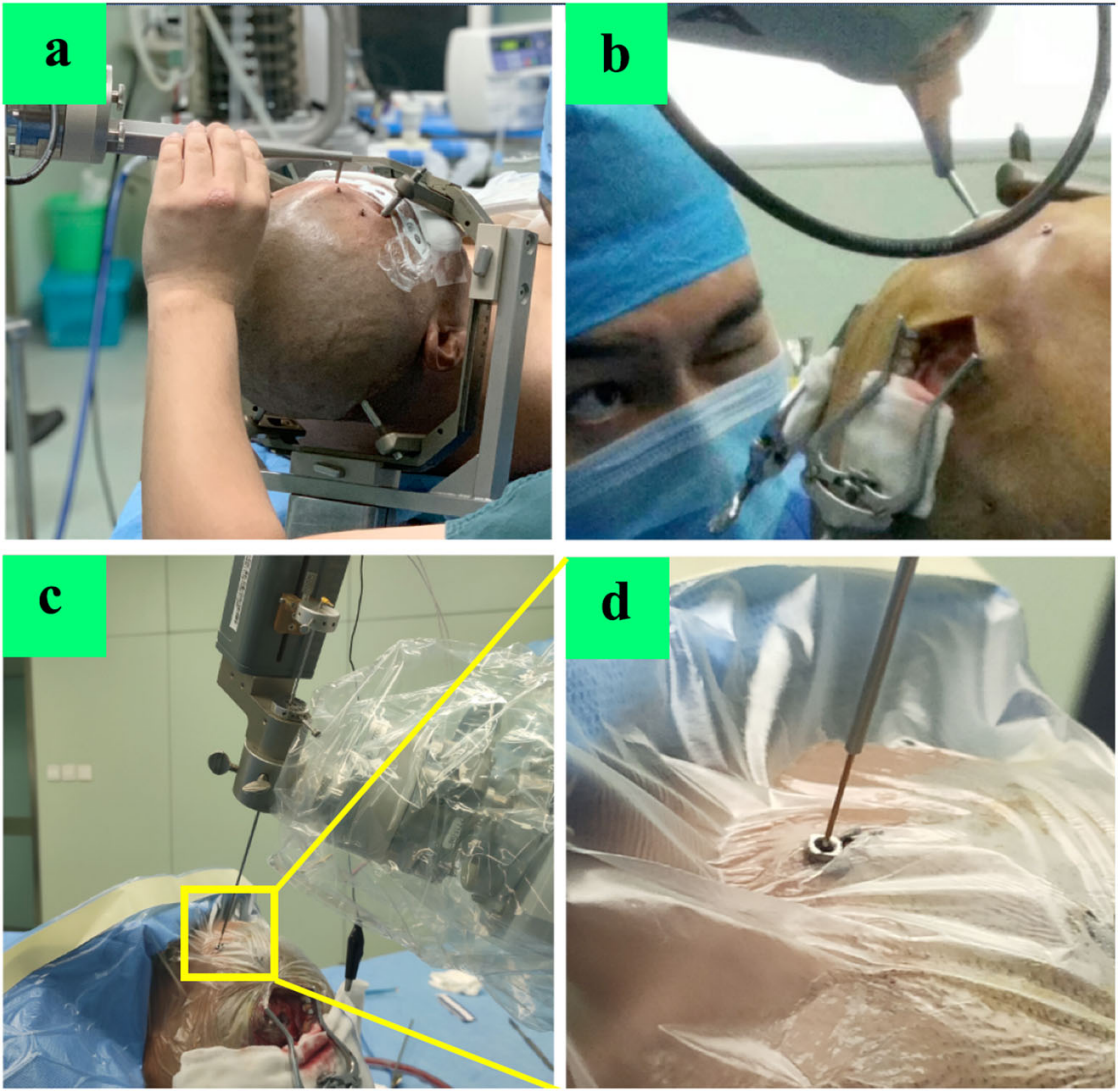
Registration and verification. (There was consent to use the photographs). **a** Preoperative registration (the first registration) of markers fixed on the skull. **b** After skin incision and skull hole drilling, intraoperative registration (the second registration) of markers. **c**, **d** Intraoperative verification of simulated target before electrode implantation.

All patients underwent preoperative MRI (Siemens MAGNETOM Verio 3T Tim) and head contrast-enhanced CT using the parameters^7^ before DBS surgery. Five metal markers (2023-VG, The ALCIS Company, Besancon, France) were fixed on the patients’ skulls before contrast-enhanced CT scanning. CT data were imported into the ROSA® (Robot of Surgery Assistant, Medtech S.A.S, France) software to create an image fused with the preoperative MRI according to three different blood vessels on the plane of the intended target. MRI and CT images were imported into the ROSA system, and a surgical trajectory was designed according to the location of the nuclei and the optimal cortical puncture point, i.e., where the cortical gyrus was closest to the dura mater, simultaneously avoiding the sulci and blood vessels (Fig. 2). The preoperative planning was led by Professor YQ Tao, and Dr. Hai Jin and Xiao Sun were responsible for inspection and verification.

### The robot-assisted neurosurgical procedure

**Fig. 3:**
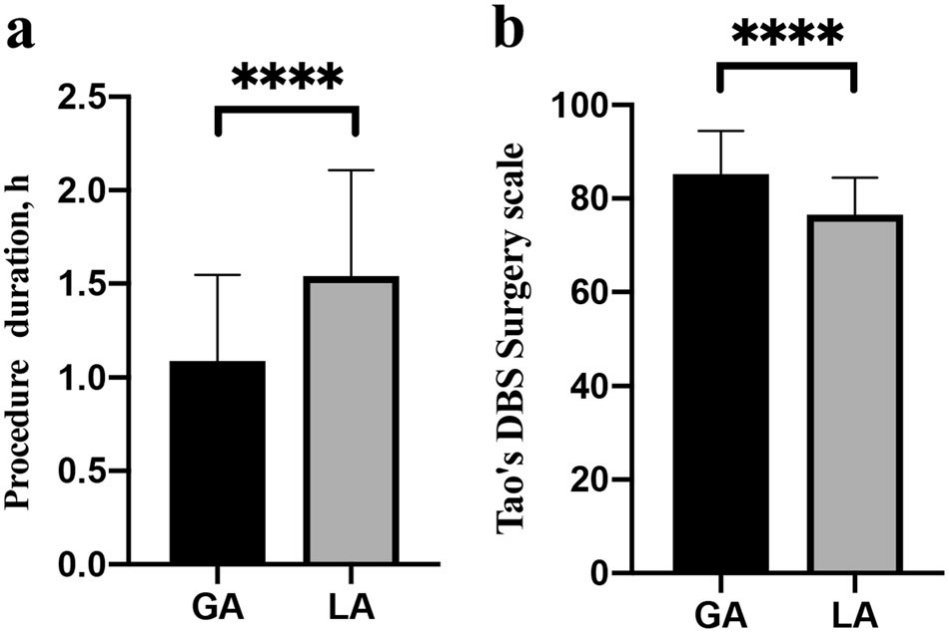
Asleep compared with awake DBS surgery. **a** Procedure duration was significantly shorter in the GA group (GA 1.09 ± 0.46 h vs. LA 1.54 ± 0.57 h, *p* < 0.0001). **b** Tao’s DBS surgery scale were significantly higher in the GA group (GA 85.2 ± 9.3 vs. LA 76.5 ± 8.0, *p* < 0.0001).

Preoperative data processing: as metal artifacts of bone markers are present in CT images, we used a modified registration method of robot-assisted DBS surgery, which can reduce the registration error and electrode vector error, as published by our center^18^. The main surgical procedure: all patients underwent surgery in the supine position with the head elevated at 10–20°. A stereotactic head frame (Leksell Modell G, Elekta Instruments, Inc., GA) was fixed to the patients’ skulls and was mainly used to fix the patients’ heads and then tightly fixed to the connecting rod of the robot. After the laser localization of the robotic manipulator to determine the position of the burr hole, the operator cut the scalp and drilled the burr hole. Preoperative registration (the first registration) of markers was performed (Fig. 3a). Next, intraoperative registration (the second registration) was performed to avoid head shift errors (Fig. 3b); this guaranteed a minimum registration error compared with the preoperative registration. Then the microelectrode on the robotic manipulator verified an implantation error of <0.4 mm (a simulated bone marker notch diameter of 0.8 mm) via simulated target verification (Fig. 3c, d). If the verification error was >0.4 mm (the edge of the bone marker notch), it was slightly corrected by adjusting the screw direction on the microelectrode thruster base to ensure the minimization of any errors before opening the dura mater. The dura mater was opened to a diameter of 2–3 mm so that one parallel steel cannula could be inserted. Then the microelectrode recording (MER) was performed intraoperatively using the alpha-omega microelectrode recording system to confirm that the target was correct. The final placement depth of the electrode was determined according to the intraoperative electrophysiological signal length or the patients’ symptom relief during surgery. When the steel cannula was pushed out after successful implantation of the electrode, the burr hole was immediately closed by bone wax and the lead was fixed. Next, bilateral pulse generators were implanted into subcutaneous pockets of the infraclavicular region. We recorded the duration of surgery beginning with scalp incision and ending with skin suture completion. Electrode implantation duration (~10 min on each side) was recorded beginning with the opening of the dura mater and ending with burr hole closure on each side.

The duration was recorded for the following procedures: (1) skin incision-stage 1 (frontal scalp incisions); (2) dural incision-first side; (3) dural incision-second side; (4) skin closure; (5) skin incision-stage 2 (Impulse Generator, IPG); and (6) skin closure. The duration of the procedure was defined as the interval between skin incision and closure (steps 1–6).

### Anesthesia

Awake surgery patients received LA with lidocaine. Asleep surgery patients received GA with endotracheal intubation. Anesthesia was induced by administration of fentanyl (1.5 µg/kg), propofol (1.5 mg/kg), and rocuronium (1 mg/kg). Desflurane inhalation was maintained during surgery and used to keep minimal alveolar concentration during scalp incision and skull hole creation. The depth of GA was adjusted by reducing minimal alveolar concentration of inhalational anesthetics to 0.6 during MER with a bispectral index (BIS) monitor (scores >70). During MER, the BIS monitor was turned off for ~10 min on each side.

### Postoperative check

Postoperative CT (Discovery CT750, GE Healthcare), with spiral scanning, 100 KV, 350 mA, and 2.0 mm slice thickness, was performed both 2 h and 1 week after surgery to assess the electrode position, pneumocephalus volume, and complications such as intracranial hemorrhage or electrode offset. The electrode fusion error compared with preoperative planning was defined as the Euclidean difference between the intended and actual trajectories of electrodes on the axial plane of the intended target. The deviation in X- and Y- coordinate vectors of the DBS lead on the intended target Z-plane were measured on each side of the fused images both 2 h and 1 week after surgery, and the total deviation (*D*) of the electrodes between the intended and actual trajectories was calculated as *D* = $\sqrt {{\mathrm{X}}^2 + {\mathrm{Y}}^2}$. The Tao’s DBS surgery scale, including electrode implantation duration, postoperative pneumocephalus volume, and electrode fusion error, was used to assess the DBS surgery.

### Clinical evaluation

The UPDRS, the Montreal Cognitive Assessment Scale, and the PD quality of life (PDQL-39) scale were all preoperatively performed on patients. The time of follow-up for postoperative neuromodulation was usually every 3 months. Most patients only required proper adjustment of the original parameters, while some patients required adjustments to the stimulating contacts. For certain patients (with dizziness, blurred vision, or unsatisfactory improvement of gait disturbance), special stimulation modes (such as cross electric pulse, variable frequency stimulation, and low frequency stimulation) were used. Furthermore, different program groups were set up for patients to use in different situations. At the 6-month follow-up, we assessed the short-term clinical efficacy of PD patients with UPDRS and UPDRS-III.

### Statistical analysis

Statistical analysis was performed using GraphPad Prism version 8.0 for Mac OS X (GraphPad Software, San Diego, CA, USA). Continuous data were expressed as the mean ± SD, and binary data were provided as percentages. The *χ*^2^ test or Fisher’s exact test were used for the binary data, and the paired *t*-tests or Wilcoxon signed-rank test were used for pre- and postoperative continuous data. The unpaired independent sample *t*-test or Mann–Whitney *U* test were used for independent continuous data (depending on whether the variable met the parametric assumptions). Analysis of covariance (ANCOVA) was performed between groups for UPDRS and UPDRS-III improvement comparison, and baseline UPDRS and the levodopa challenge were selected as covariates. The Bonferroni correction was performed for multiple comparisons. *P* < 0.05 (two-tailed) was considered statistically significant.

### Statement regarding ethics committee approval and patient consent

This study was approved by the local ethics committee of the General Hospital of Northern Theater Command and was conducted in accordance with the Declaration of Helsinki. All participants provided written informed consent to take part in this study, and the participants in Fig. 3 provided consent for the photo to be published.

### Reporting summary

Further information on research design is available in the Nature Research Reporting Summary linked to this article.

## Supplementary information

Reporting Summary

## Data Availability

The datasets generated and analyzed during the current study are available from the corresponding author upon reasonable request.
